# Malignant Pleural Mesothelioma Outcomes in the Era of Combined Platinum and Folate Antimetabolite Chemotherapy

**DOI:** 10.1155/2015/590148

**Published:** 2015-03-02

**Authors:** Mathieu D. Saint-Pierre, Christopher Pease, Hamid Mithoowani, Tinghua Zhang, Garth A. Nicholas, Scott A. Laurie, Paul Wheatley-Price

**Affiliations:** ^1^Department of Medicine, University of Ottawa, 501 Smyth Road, Ottawa, ON, Canada K1H 8L6; ^2^Department of Medicine, Queen's University, 102 Stuart Street, Kingston, ON, Canada K7L 2V6; ^3^The Ottawa Hospital Research Institute, 725 Parkdale Avenue, Ottawa, ON, Canada K1Y 4E9

## Abstract

*Introduction*. Malignant pleural mesothelioma (MPM) is associated with a poor prognosis. Palliative platinum-based chemotherapy may help to improve symptoms and prolong life. Since 2004, the platinum is commonly partnered with a folate antimetabolite. We performed a review investigating if survival had significantly changed before and after the arrival of folate antimetabolites in clinical practice. *Methods*. All MPM patients from January 1991 to June 2012 were identified. Data collected included age, gender, asbestos exposure, presenting signs/symptoms, performance status, histology, stage, bloodwork, treatment modalities including chemotherapy, and date of death or last follow-up. The primary endpoint was overall survival. Cox models were applied to determine variables associated with survival. *Results*. There were 245 patients identified. Median overall survival for all patients was 9.4 months. After multivariate analysis, performance status, stage, histology, leucocytosis, and thrombophilia remained independently associated with survival. Among all patients who received chemotherapy, there was no difference in overall survival between the periods before and after folate antimetabolite approval: 14.2 versus 13.2 months (*P* = 0.35). Specifically receiving combined platinum-based/folate antimetabolite chemotherapy did not improve overall survival compared to all other chemotherapy regimens: 14.1 versus 13.6 months (*P* = 0.97). *Conclusions*. In this review, we did not observe an incremental improvement in overall survival after folate antimetabolites became available.

## 1. Introduction

Malignant pleural mesothelioma (MPM) is a relatively uncommon malignancy arising from the pleura of the lung. In the United States, incidence in 2011 was estimated to be approximately 1 case per 100,000 population [1]. In Canada, the incidence of mesothelioma was 1.2 per 100,000 in 2010 [2]. Exposure to asbestos is the major risk factor in developing MPM, and there is often a lag-time of many decades between asbestos exposure and development of the disease [3, 4]. Peak incidence in the United States appears to have occurred in the 1990s and seems to be declining slowly. However, peak incidence has not yet occurred elsewhere in the developed world nor in the developing world. As such, the worldwide burden of disease is expected to increase as asbestos continues to be used as a building material in many countries [4].

Currently, the prognosis for MPM patients is guarded. Most patients present with advanced disease at the time of diagnosis, and MPM is almost universally fatal. Median survival has been estimated to be between 9 and 17 months across all stages [5]. Independent risk factors with prognostic value have been identified in the literature. The Cancer and Leukaemia Group B (CALGB) identified 10 patient characteristics including laboratory, clinical, and pathological factors associated with poor prognosis and used these to stratify patients into 6 prognostic groups [6]. This association has subsequently been validated [7]. The European Organisation for Research and Treatment of Cancer (EORTC) has also previously developed a validated model which divides patients into a low or high risk group based on the number of poor prognostic factors present [8, 9].

Treatment options for MPM are limited. Conducting randomized clinical trials is difficult due to the relatively low incidence of the disease. Furthermore, there is little homogeneity between studies which makes drawing conclusions about its treatment difficult [5]. Commonly used palliative chemotherapy is a platinum-based agent combined with a folate antimetabolite, either pemetrexed or raltitrexed. Both have shown an advantage in overall survival when used in combination with cisplatin when compared to cisplatin monotherapy [10, 11]. Of note, there are no randomized studies that have demonstrated improved survival with chemotherapy compared to best supportive care alone [12]. Patients who do present with more limited disease may be considered for trimodality therapy in some specialized centres. This consists of induction chemotherapy, an extrapleural pneumonectomy, and postoperative radiation, but the role of this approach is controversial [13].

We performed a 21-year single institution retrospective review of all cases of MPM to determine if overall survival has improved since folate antimetabolite agents became available in routine clinical practice.

## 2. Materials and Methods 

After obtaining ethics approval from our local institution, all patient charts with pathology proven malignant pleural mesothelioma (MPM) were identified between January 1991 and June 2012. The Ottawa Hospital is a tertiary care academic hospital which is the sole provider of thoracic surgery, medical, and radiation oncology services to a mixed urban and rural population of approximately 1.4 million. Patients with malignant mesothelioma of peritoneal or tunica vaginalis origin were excluded. The following variables were collected: age, sex, International Mesothelioma Interest Group (IMIG) stage, histological subtype, Eastern Cooperative Oncology Group (ECOG) performance status, presenting symptoms and signs, weight loss ≥5%, leukocytosis defined as WBC count ≥ 8.7 × 10^9^/L, thrombocytosis defined as platelet count > 400 × 10^9^/L, hemoglobin < 146 × 10^9^/L, and LDH > 500 IU/L. Cutoffs for laboratory values were chosen based on the CALGB prognostic scoring system [6]. Information on treatment received, including type of chemotherapy, was also collected.

The primary outcome was overall survival, which was defined as time from the diagnosis of MPM to the date of death by any cause or of last follow-up. Overall survival was compared between MPM patients having received chemotherapy before and after the approval of pemetrexed in Canada on May 21st, 2004. A second analysis was performed comparing those who obtained at least one cycle of platinum-based chemotherapy in conjunction with a folate antimetabolite versus patients who received other chemotherapy regimens. Patient characteristics and treatment modalities significantly associated with the primary outcome were identified by application of Cox proportional hazard models to each collected variable. Hazard ratios, their 95% confidence intervals, and *P* values were estimated. Those that were statistically significant for overall survival (defined as *P* < 0.05) were included in the multivariable analysis to determine if they remained statistically significant for our primary outcome. All statistical analysis was carried out using SAS version 9.2 (SAS Institute Inc.).

## 3. Results 

### 3.1. Baseline and Clinical Characteristics

There were 245 MPM patients identified during the defined study period. As expected, the majority were men (87%), and about half had a known asbestos exposure. Histology was epithelial (63%), sarcomatoid (14%), mixed (11%), or unknown (12%). Common presentations were dyspnea (76%) and pleural effusion (73%). Baseline clinical characteristics are outlined in Table 1.

### 3.2. Treatment Received

One hundred thirty-nine patients (57%) received at least one course of chemotherapy. The most commonly used first-line chemotherapy regimens were platinum-based chemotherapy with pemetrexed (31%), cisplatin/doxorubicin with or without tamoxifen (23%), platinum-based chemotherapy with gemcitabine (13%), and gemcitabine monotherapy (11%). A full list of first-line systemic therapies is shown in Table 2. There were 90 patients (37%) who underwent at least one course of radiation therapy, 7 of whom received this as adjuvant therapy and 83 as palliative therapy. Fifty-seven patients (23%) underwent pleurodesis, 20 (8%) underwent extrapleural pneumonectomy, and 12 (5%) had pleurectomy.

### 3.3. Survival

**Figure 1:**
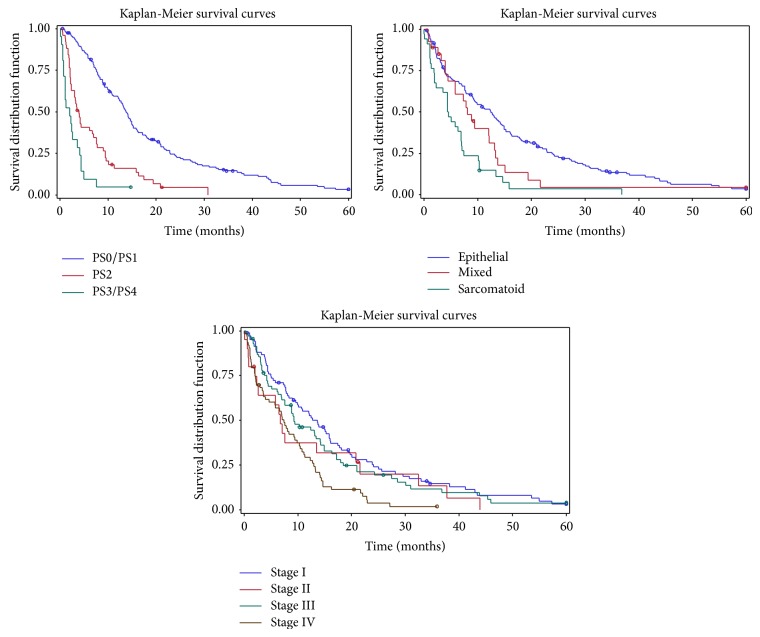
Unadjusted Kaplan-Meier curves comparing survival stratified by performance status, histology, and stage.

At the time of last follow-up, 3 patients were alive without evidence of MPM, 18 were alive with MPM, and 224 were deceased. Median overall survival was 9.4 months in all MPM patients (Table 3). Kaplan-Meier survival curves are shown for patients categorized by performance status, histology, and stage (Figure 1). In univariate analysis, factors associated with shorter survival were increasing age, poor performance status, nonepithelial histology, stage, weight loss ≥5%, leucocytosis (≥8.7), and thrombophilia (>400). Factors associated with longer survival were extrapleural pneumonectomy, chemotherapy, and radiation therapy. Performance status, histology, stage, leucocytosis, thrombophilia, chemotherapy, and radiation therapy remained independently associated with overall survival after multivariate analysis (Table 4).

### 3.4. Effect of Chemotherapy on Survival

Receipt of any chemotherapy was associated with longer overall survival in both univariate and multivariate analyses (see Table 4).

**Figure 2:**
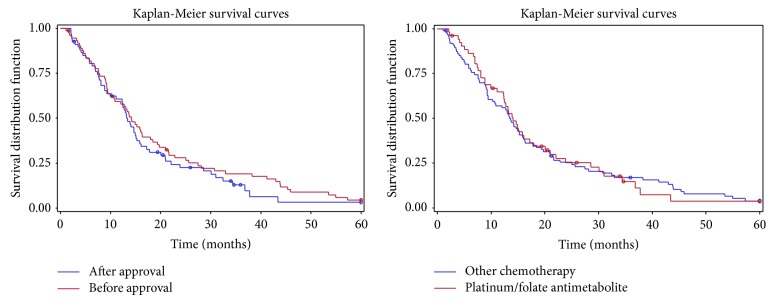
Unadjusted Kaplan-Meier curves comparing survival of malignant pleural mesothelioma (MPM) patients diagnosed before and after the approval of pemetrexed as well as patients having received platinum-based chemotherapy with pemetrexed versus all other chemotherapeutic regimens.

Out of the 139 patients who received chemotherapy, 72 were diagnosed prior to the approval of pemetrexed in Canada on May 21st, 2004, and 67 after this date. Thus, 51% of patients diagnosed before its approval received chemotherapy compared to 64% afterwards. There was no difference in overall survival between these 2 groups (overall survival 14.2 versus 13.2 months, resp., *P* = 0.35). A Kaplan-Meier survival graph was also constructed to compare the two groups (Figure 2).

Of the 67 patients treated after May 21st, 2004, 52 patients (78%) received at least one cycle of either cisplatin/pemetrexed or carboplatin/pemetrexed as part of their chemotherapeutic regimens, with a median of 3 cycles administered. No patients at our institution received a combination regimen including raltitrexed. There were 2 patients receiving a study drug (sunitinib) in addition to cisplatin/pemetrexed who were excluded from this group of 52 patients. 43 (83%) received platinum/pemetrexed as first-line therapy whereas the other 9 (17%) received it as second- or third-line therapy. The majority had an ECOG performance status of 0 or 1 (87%) and were of epithelial histology (69%). Furthermore, 42% had stage I disease.

The overall survival of the platinum/pemetrexed group was compared to that of patients who received any other chemotherapeutic regimen both before and after the arrival of folate antimetabolites. Prognostic patient characteristics such as performance status, histology, and stage were not significantly different between these two groups (*P* > 0.05). There was no difference seen in overall survival (14.1 versus 13.6 months, resp., *P* = 0.97) (Figure 2).

## 4. Discussion

Over a 21-year period, we collected data on 245 patients with a histological diagnosis of MPM. The relatively small number of patients reflects the rarity of MPM. Our demographics are similar to the International Association for the Study of Lung Cancer (IASLC) database of over 3000 patients with MPM, supporting the generalizability of our findings. In particular, the majority of patients were male (87% in our study versus 79% in the database) and presented in the seventh decade of life (median age 68 versus 63). The relative prevalence of histological subtypes was also similar with the epithelial subtype being the most common histology (63% versus 62%) [14]. Notably, more patients presented with stage I disease in our cohort (34% versus 11%). This could be related to variability in staging systems used across the study period. Consistent with previous studies, patient variables such as performance status, histology, and stage were shown to be independently associated with survival [6, 7]. While there is clear separation of curves in the Kaplan-Meier graphs (Figure 1) reporting performance status and histology, the more clustered lines in the Kaplan-Meier graph by stage emphasize the point that stage is difficult to determine accurately in MPM.

Chemotherapy receipt for MPM did increase over time at our institution. Those who received chemotherapy lived significantly longer than those patients who did not. In an attempt to reduce selection bias, results were adjusted for identified prognostic variables and this statistically significant difference remained. In contrast, a randomized controlled trial in 2008 compared MVP (mitomycin, vinblastine, and cisplatin) or vinorelbine chemotherapy to best supportive care but failed to demonstrate a statistically significant difference in overall survival [12]. Only one patient received first-line single agent vinorelbine in our study population and none received MVP. This suggests that differences in the choice of chemotherapy regimens may explain the discrepancy in findings.

In the landmark study by Vogelzang et al., cisplatin and pemetrexed led to an objective response rate of 41% and median survival of 12.1 months, compared to 17% and 9.3 months in patients treated with cisplatin alone [11]. Similar results were seen in the smaller cisplatin/raltitrexed study published by van Meerbeeck et al.; however we did not identify any patients who received this regimen in our centre [10]. There are multiple smaller phase II studies that have investigated other platinum doublets, but no randomized studies comparing cisplatin/pemetrexed with other platinum doublet combinations [15–17]. In order to assess whether the addition of pemetrexed had any significant impact on survival in a real-world setting, we split our cohort between those treated before and after pemetrexed was approved in Canada. After this date, the majority (78%) of patients who obtained chemotherapy in our review received pemetrexed as part of a platinum-based combination regimen. Our analysis however failed to show a statistically significant improvement in survival after pemetrexed approval. Furthermore, when specifically comparing patients who received platinum/pemetrexed to those who did not, we again failed to show a statistically significant difference in overall survival. This highlights the difficulties of translating results seen in clinical trials to real-world practice. Aside from this, some patients who were accepted in our cohort may not have been included in the Vogelzang et al. trial. This study only included patients with satisfactory performance status (Karnofsky score at least ≥70, 54% being 90–100) and excluded patients who had received any prior chemotherapy. They also excluded patients with limited disease who were candidates for possible curative surgery. We feel that these more stringent eligibility criteria would likely leave out a significant proportion of MPM patients currently being treated with chemotherapy, although the majority of those who received platinum/pemetrexed in our review would have been included in the landmark trial. It is thus unlikely that differences in inclusion criteria would explain the divergent results seen between the 2 trials. As well, the cisplatin/pemetrexed cohort had a median of 6 cycles in the randomized controlled trial compared to 3 cycles in our population. The selection of patients with good performance status could again help to explain in part this difference. An alternative explanation may be that platinum-based agents provide benefit when partnered with many other cytotoxic agents as has been suggested by several phase II trials [15–17]. A previously published retrospective study did not show a significant difference in survival between MPM patients who received platinum-based chemotherapy with either pemetrexed or gemcitabine [18].

The strengths of this study include its relatively large number of patients given the rarity of this disease and the adjustment of survival data by prognostic variables. To our knowledge, this represents one of the largest retrospective analyses in the post folate antimetabolite era. Our study is however limited by its retrospective nature, where not all data variables are systematically recorded at the time of the clinical encounter. Generalizability may be limited given that analysis took place at a single institution despite broad similarities to previously published databases [14].

In conclusion, this study reports the results of a comprehensive analysis of a large tertiary care centre's experience with MPM. Our study found increased survival among patients who received any chemotherapy compared to those who did not. However, it did not demonstrate improved survival in MPM patients after the introduction into clinical practice of folate antimetabolites, specifically pemetrexed, as a partner drug to platinum-based chemotherapy.

## Figures and Tables

**Table 1:** Baseline demographic data.

Malignant pleural mesothelioma (*n*)	245 patients
Age, median (range), y	68 (21 to 88)
Gender, number (%)	
Men	213 (86.9%)
Women	32 (13.1%)
Known asbestos, number (%)	
Yes	123 (50.2%)
No	122 (49.8%)
Eastern Cooperative Oncology Group (ECOG) performance status, number (%)	
0	37 (15.1%)
1	117 (47.8%)
2	51 (20.8%)
3	19 (7.8%)
4	2 (0.8%)
Unknown	19 (7.8%)
Initial staging, number (%)	
I	84 (34.3%)
II	20 (8.2%)
III	69 (28.2%)
IV	63 (25.7%)
Unknown	9 (3.7%)
Histology, number (%)	
Epithelial	154 (62.9%)
Sarcomatoid	34 (13.9%)
Mixed	27 (11%)
Unknown	30 (12.2%)
Laterality, number (%)	
Left	97 (39.6%)
Right	138 (56.3%)
Bilateral	5 (2%)
Unknown	5 (2%)
Presenting symptoms and signs, number (%)	
Dyspnea	186 (75.9%)
Pleural effusion	179 (73.1%)
Chest pain	118 (48.2%)
Weight loss (≥5%)	89 (36.3%)
Cough	71 (29%)
Dysphagia	7 (2.9%)
Pericardial effusion	5 (2%)

**Table 2:** First-line chemotherapeutic regimens.

First-line chemotherapy	*n* (%)	Before pemetrexed approval	After pemetrexed approval
Platinum-based chemotherapy with pemetrexed	43 (30.9%)	0	43
Cisplatin/doxorubicin with or without tamoxifen	32 (23.0%)	32	0
Platinum-based chemotherapy with gemcitabine	18 (12.9%)	15	3
Gemcitabine	15 (10.8%)	15	0
Gefitinib	10 (7.2%)	0	10
Other regimens (each regimen representing <5%)	21 (15.1%)	10	11

**Table 3:** Overall survival by performance status, histology, and stage.

		Patients (*n*)	Median OS (months)	95% confidence interval	
				Lower	Upper
All patients		245	9.4	7.8	12.0

Eastern Cooperative Oncology Group (ECOG) performance status	0, 1	154	13.7	12.1	15.1
	2	51	4.0	2.3	6.7
	3, 4	21	2.0	0.8	3.6

Histology	Epithelial	154	12.5	9.3	14.4
	Sarcomatoid	34	4.5	2.0	6.9
	Mixed	27	8.1	4.5	12.1

Stage	I	84	13.6	9.0	16.0
	II	20	6.7	2.0	20.8
	III	69	9.3	7.0	14.2
	IV	63	7.4	4.0	9.9

**Table 4:** Multivariate Cox regression analysis of patient and treatment factors.

		Patients (*n*)	HR	95% confidence interval		*P* value (Cox model)
				Lower	Upper	
Age at diagnosis	<70 years old	128	Ref.			
	≥70 years old	117	1.20	0.84	1.71	0.33

Eastern Cooperative Oncology Group (ECOG) performance status	0, 1	154	Ref.			
	2	51	2.41	1.56	3.74	<0.01
	3, 4	21	4.84	2.44	9.58	<0.01

Histology	Epithelial	154	Ref.			
	Sarcomatoid	34	2.18	1.33	3.55	<0.01
	Mixed	27	2.10	1.25	3.55	<0.01

Stage	I	84	Ref.			
	II	20	1.08	0.57	2.05	0.81
	III	69	1.54	1.03	2.30	0.04
	IV	63	2.04	1.35	3.09	<0.01

Symptoms	Weight loss <5%	156	Ref.			
	Weight loss ≥5%	89	1.44	0.98	2.12	0.06

Bloodwork	White blood cell count <8.7	118	Ref.			
	White blood cell count ≥8.7	109	1.55	1.08	2.25	0.02
	Platelets ≤400	171	Ref.			
	Platelets >400	55	1.64	1.11	2.42	0.01

Extrapleural pneumonectomy	Yes	20	Ref.			
	No	225	1.57	0.80	3.08	0.19

Chemotherapy	Yes	139	Ref.			
	No	106	2.32	1.59	3.39	<0.01

Radiation therapy	Yes	90	Ref.			
	No	155	1.60	1.13	2.27	<0.01
